# A Nanoparticle-Based Immunoassay on Facemasks for Evaluating Neutrophilic Airway Inflammation in COPD Patients

**DOI:** 10.3390/bios15050323

**Published:** 2025-05-19

**Authors:** Bartomeu Mestre, Nuria Toledo-Pons, Andreu Vaquer, Sofia Tejada, Antonio Clemente, Amanda Iglesias, Meritxell López, Ruth Engonga, Sabina Perelló, Borja G. Cosío, Roberto de la Rica

**Affiliations:** 1 Multidisciplinary Sepsis Group, Health Research Institute of the Balearic Islands (IdISBa), 07120 Palma de Mallorca, Spain; bartomeu.mestrec@idisba.es (B.M.); roberto.delarica@idisba.es (R.d.l.R.); 2 Inflamación, Reparación y Cáncer en Enfermedades Respiratorias (I-RESPIRE) Group, Health Research Institute of the Balearic Islands (IdISBa), 07120 Palma de Mallorca, Spain; nuria.toledo@ssib.es (N.T.-P.); amanda.iglesias@ssib.es (A.I.); meritxell.lopez@ssib.es (M.L.); ruth.engonga@ssib.es (R.E.); sabina.perello@ssib.es (S.P.); borja.cosio@ssib.es (B.G.C.); 3 Department of Respiratory Medicine, Hospital Universitario Son Espases, 07120 Palma de Mallorca, Spain; 4 Department of Chemistry, University of the Balearic Islands, 07122 Palma de Mallorca, Spain; 5 Group of Innovation in Immunopathology of Infections (GTERi), Health Research Institute of the Balearic Islands (IdISBa), 07120 Palma de Mallorca, Spain; antonio.clemente@idisba.es; 6 Centro de Investigación Biomédica en Red de Enfermedades Infecciosas, Instituto de Salud Carlos III (CIBERINFEC), 28029 Madrid, Spain; 7 Centro de Investigación Biomédica en Red de Enfermedades Respiratorias, Instituto de Salud Carlos III (CIBERES), 28029 Madrid, Spain

**Keywords:** chronic obstructive pulmonary disease, breath, myeloperoxidase, exacerbation, biosensor

## Abstract

Patients with chronic obstructive pulmonary disease (COPD) often experience acute exacerbations characterized by elevated neutrophilic inflammation in the lungs. Currently, this condition is diagnosed through visual inspection of sputum color and volume, a method prone to personal bias and unsuitable for patients who are unable to expectorate spontaneously. In this manuscript, we present a novel approach for measuring and monitoring exhaled myeloperoxidase (MPO), a biomarker of neutrophilic airway inflammation, without the need for sputum analysis. The method involves analyzing an unmodified surgical facemask worn by the patient for 30 min using biosensing decals that transfer antibody-coated nanoparticles. These colloids specifically interact with MPO trapped by the facemask in a dose-dependent manner, enabling the quantification of MPO levels, with a dynamic range up to 3 · 10^1^ µg·mL^−1^. The proposed diagnostic approach successfully differentiated patients with acute exacerbations from stable patients with 100% sensitivity and specificity. Healthy individuals also showed significantly lower MPO levels compared to COPD patients. Our results suggest that facemask analysis could be a non-invasive diagnostic tool for airway diseases, particularly in patients unable to expectorate.

## 1. Introduction

Chronic obstructive pulmonary disease (COPD) is a debilitating condition that causes shortness of breath in over 480 million people worldwide [1]. It is characterized by chronic airway inflammation and progressive airflow limitation [2,3]. Patients experience acute exacerbations, defined as “an event in the natural course of the disease characterized by a worsening of the patient’s baseline dyspnea, cough and/or sputum beyond day-to-day variability sufficient to warrant a change in management” [4]. These episodes are primarily triggered by respiratory infections (viral or bacterial), and environmental factors such as air pollution [5], which amplify inflammation and promote neutrophil recruitment to the airways [6]. Poorly managed exacerbations are associated with irreversible loss of lung function; therefore, early detection and treatment are essential to improve patient prognosis [5,7].

Several pulmonary biomarkers have been proposed to identify exacerbations [8,9], including some that indicate neutrophilic inflammation [10]. However, using lung biomarkers for management poses challenges, as sputum (considered the most representative sample of the lower respiratory tract) is not always readily available, since not all patients can spontaneously expectorate [11]. Thus, it is desirable to develop an alternative method that circumvents the need for sputum analysis when evaluating neutrophilic airway inflammation. Such a method could enhance COPD management by allowing early exacerbation detection and treatment, thereby preventing hospitalizations and irreversible lung damage.

Neutrophils play a key role in the pathogenesis of COPD, particularly during acute exacerbations. Upon activation, neutrophils release various pro-inflammatory mediators, including myeloperoxidase (MPO), an enzyme stored in their primary granules. MPO catalyzes the production of reactive oxygen species and hypochlorous acid, both of which contribute to airway tissue damage and inflammation. Elevated MPO levels have been associated with neutrophilic inflammation and the severity of COPD exacerbations, making it a potential biomarker for airway inflammation [8].

In this article, we propose a novel, non-invasive method to assess neutrophilic inflammation in the airways of COPD patients by detecting MPO [8] on surgical facemasks worn by patients. These are standard, unmodified surgical masks that trap aerosols exhaled during breathing. We hypothesized that the amount of MPO detected in the mask may correlate with neutrophilic airway inflammation. This approach overcomes limitations of exhaled breath condensate analysis [9,10,11], which have been criticized for inconsistent results due to variable sample dilution [12,13]. Moreover, exhaled breath condensate collection requires nursing supervision, thereby increasing healthcare burden. In contrast, surgical facemasks trap undiluted aerosols and can be worn without supervision, for instance, in waiting rooms. Furthermore, since many hospitals already provide these masks to control the spread of respiratory pathogens, this strategy could be easily integrated into current clinical workflows.

**Figure 1:**
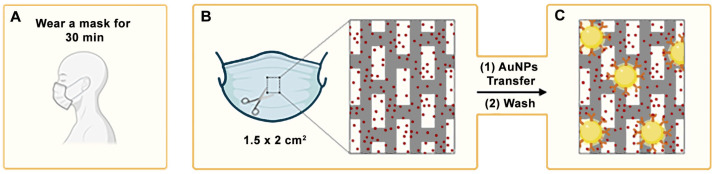
A schematic representation of the main steps required to diagnose neutrophilic inflammation in airways with the proposed methodology: (**A**) the facemask becomes imbued with myeloperoxidase as the patient breathes; (**B**) the intermediate polypropylene layer is isolated; (**C**) antibody-coated nanoparticles (represented as the yellow circles) are transferred from a reservoir in a paper decal. After washing, nanoparticles bound by antibody–antigen interactions generate a colored spot on the facemask. AuNPs: gold nanoparticles.

In our approach, facemask sample collection is combined with rapid detection using a nanoparticle-based immunoassay (Figure 1) [14,15,16]. To detect MPO, the polypropylene layer of a facemask worn by a patient is isolated (Figure 1A,B). A paper decal containing antibody-coated nanoparticles is then pressed against the mask. These nanoprobes specifically bind to MPO adhered to the facemask via antibody–antigen interactions (Figure 1C). After 5 min, excess reagents are washed away, leaving behind a colored spot whose pixel intensity correlates with the concentration of the target analyte trapped in the facemask. The complete immunoassay takes less than 10 min, and the resulting signal can be quantified using image-processing software such as ImageJ (version 1.54 g) or a smartphone application [16].

In contrast to other facemask-based analyte detection methods, our approach does not require embedding sensors in the facemask itself [17,18,19]. As a result, sample collection can be performed using a standard surgical facemask, making it a low-cost and universally applicable method for airway sampling. Previous studies using this platform have demonstrated that respiratory biomarkers and pathogens can be detected in facemasks worn for just 30 min. Wear times of 10, 30, and 120 min were systematically evaluated to assess the impact of mask usage duration on detection sensitivity [14,15,16]. A 10-min wear time reduced detection sensitivity, while a 120-min duration did not improve sensitivity and also compromised measurement reproducibility. Based on these findings, a 30-min wear time was selected as the optimal duration.

In this manuscript, our method can distinguish COPD patients experiencing an exacerbation from those in a stable phase of the disease, achieving 100% sensitivity and specificity. It also differentiates stable COPD patients from healthy volunteers, as the former display higher levels of airway inflammation. This outstanding performance, combined with a non-invasive sampling process that does not require expectoration, highlights the potential of our approach as a promising tool for monitoring neutrophilic inflammation in the airways of COPD patients.

## 2. Materials and Methods

### 2.1. Reagents

Gold (III) chloride hydrate and sodium citrate tribasic dehydrate (Sigma-Aldrich, St. Louis, MO, USA) were used for nanoparticle synthesis. Poly(ethylene glycol) 2-mercaptoethyl ether acetic acid (thiol-PEG-acid, Mw ≈ 2100), Poly(sodium 4-styrenesulfonate) (Mw ≈ 70.000, 30% solution), Tween-20 and N-Hydroxysulfosuccinimide sodium salt (sulfo-NHS) were sourced from Sigma-Aldrich (MO, USA). Piece™ EDC, No-Weigh™ was purchased from Thermo Scientific (Waltham, MA, USA). A rabbit polyclonal MPO antibody was acquired from Bioss Antibodies (Woburn, MA, USA), and human MPO from polymorphonuclear leukocytes was provided by Sigma-Aldrich. Whatman filter paper #1 was obtained from Cytiva (Washington, DC, USA), and blotting paper was obtained from Bio-Rad (Hercules, CA, USA). Bovine serum albumin (BSA, protease-free) was supplied by VWR Chemicals (Radnor, PA, USA). Phosphate-buffered saline (PBS) was prepared at pH 7.4; PBS-Tween refers to PBS supplemented with 0.1% Tween-20.

### 2.2. Antibody-Coated Nanoparticles

Gold nanoparticles with an average diameter of 40 nm were synthesized and modified with avidin according to a previously published method [20]. Briefly, sodium citrate (57 mg) was added to a boiling 250 mL solution containing 49 mg of gold (III) chloride trihydrate under agitation. The solution turned burgundy red, indicating the formation of gold nanoparticles with a localized surface plasmon resonance (LSPR) at 527 nm. The colloids were stabilized with polyethylene glycol ending in thiol and carboxylate groups (SH-PEG-COOH). Carboxylate groups were then activated with EDC and sulfo-NHS, followed by conjugation of avidin through peptide bond formation. Unreacted groups were capped with glycine and BSA. The resulting avidin-decorated nanoparticles were then modified with 25 μg·mL^−1^ of biotinylated anti-MPO and kept at 4 °C until use. Control nanoparticles were obtained by adding biotinylated BSA instead of anti-MPO. 

### 2.3. Manufacturing of Nanoparticle Reservoirs

Whatman paper #1 was infused with PSS following a previously published procedure [14]. Anti-MPO nanoparticles were spotted on the PSS-coated films using a Hamilton syringe (Reno, NV, USA) (0.5 µL per spot). The resulting paper decals, containing nanoparticle reservoirs, were cut into 0.5 × 0.5 cm^2^ pieces.

### 2.4. Calibration Experiments

Facemasks were cut into 1.5 × 2 cm^2^ squares for the calibration experiments. The polypropylene layer was isolated, and MPO solutions at different concentrations were sprayed twice onto the facemask pieces from a distance of 5 cm, then allowed to dry for 30 min [16]. Each facemask piece was placed onto three 1.8 × 1.8 cm^2^ squares of blotting paper. After adding the blocking solution (PBS-BSA) onto the blotting paper, the nanoparticle reservoir was placed on top of the facemask piece, and the entire assembly was pressed together for 5 min. A washing step was then performed by adding 150 µL of PBS-Tween to the blotting paper layers and applying gentle pressure to the reservoir three times.

Colorimetric signals were evaluated by scanning the assays using an MCF-1910W scanner (Nagoya, Aichi, Japan), followed by quantification of pixel intensity within the colored spot using ImageJ (version 1.54 g), with background signal subtracted.

### 2.5. Analysis of Facemasks Worn by Patients

Facemasks were collected after being worn by patients for 30 min. From each mask, four 1.5 × 2 cm^2^ squares were cut from the central area, and the polypropylene layer was isolated for analysis. The assay steps including blocking, nanoparticle transfer, and washing were then performed as described in the previous section (Figure 1). These steps required approximately 5 min in total. The facemask pieces were subsequently scanned and analyzed using ImageJ software to quantify the resulting colorimetric signal. This analysis step took approximately 2 min and could be further shortened by using a dedicated mobile application [21]. The total time from facemask collection to result interpretation was under 40 min, with less than 10 min required after sample retrieval.

### 2.6. Study Design

This was an observational, prospective, pilot study that included consecutive patients with COPD recruited from the outpatient COPD clinics of the pneumology department of the Hospital Universitario Son Espases between February 2023 and June 2024.

### 2.7. Ethics Declarations

The study protocol was approved by the Ethics Committee of the Balearic Islands (Mallorca, Spain) and registered under number IB 5361/23 PI, in accordance with the Declaration of Helsinki. Informed consent for the collection of exhaled breath samples, provided by the Biobank Platform of the Instituto de Investigación Sanitaria de las Islas Baleares (IdISBa), was obtained from all patients by the attending physician or a trained study staff member before any study procedures. All experiments were performed in compliance with relevant guidelines and regulations.

### 2.8. Inclusion and Exclusion Criteria

Patients aged ≥18 years with a diagnosis of COPD according to international guidelines [4] and managed in specific COPD outpatient respiratory clinics were eligible for inclusion. The exclusion criteria were patients with cancer, acute or chronic inflammatory/autoimmune diseases, drug consumption, and withdrawal or informed consent.

Exacerbation was defined, according to Anthonisen et al. [22], as increased dyspnea, sputum production, and sputum purulence. Spontaneously expectorated sputum samples from patients with exacerbations were collected and cultured. Gram-staining of the most purulent areas of the sputum was performed to assess leukocytes and epithelial cell content. Samples classified as Murray–Washington criteria IV (10–25 epithelial cells and >25 leukocytes per field) or V (≤10 epithelial cells and >25 leukocytes per field) were considered representative of distal airways secretions and processed for culture [23]. Clinical stability was defined as the absence of exacerbations at the time of facemask collection. Healthy subjects without COPD were recruited as controls.

### 2.9. Clinical Data Collection

Demographic data collected included age, sex, allergies, BMI, and smoking status. Clinical variables included lung function parameters (FEV1, FEV1/FVC, DLCO), arterial blood gas tests (PaO_2_, PaCO_2_), GOLD classifications [5,24], inflammatory markers (white blood cell count, neutrophil and eosinophil counts, and C-reactive protein levels), the presence of bronchiectasis, and colonization by *Pseudomonas aeruginosa* or other pathogens. Dyspnea severity was assessed using the modified Medical Research Council (mMRC) dyspnea scale [25]. Additionally, data from sputum Gram-staining and culture results were recorded.

### 2.10. Statistical Analyses

The data were entered into a Microsoft Office Excel 2010 spreadsheet by designated personnel. Statistical analyses were conducted using GraphPad Prism 9 and RStudio version 2022. Statistical significance was considered at *p*-value ≤ 0.05. Baseline characteristics of the study population are reported. The Kolmogorov–Smirnov test was used to assess the normality of data distributions. Continuous variables are reported as means ± standard deviations (SDs) for normally distributed data, or as medians with interquartile ranges (IQR) for non-normally distributed data. Categorical variables are presented as absolute frequencies and percentages. Two-group comparisons were performed using independent samples *t*-tests for parametric data or Mann–Whitney U tests for non-parametric data. Categorical variables were analyzed using the chi-square test. Comparisons involving three groups were conducted using one-way analysis of variance (ANOVA).

### 2.11. Data Availability

The data supporting the findings of this study are not publicly accessible due to confidentiality and sensitivity concerns but may be obtained from the corresponding author (ST) upon reasonable request. All data are securely stored in a controlled-access repository at the Pneumology Department of the Hospital Universitario Son Espases, Balearic Islands, Spain.

## 3. Results and Discussion

**Figure 2:**
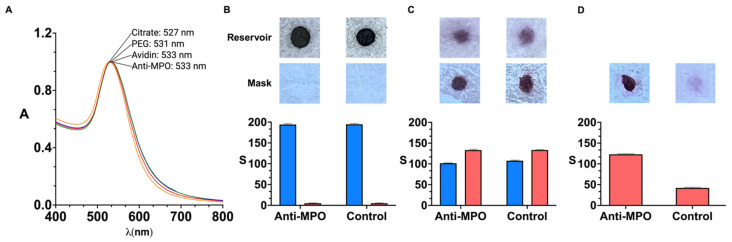
Characterization of detection system; vis-NIR spectrum of gold nanoparticles after each modification step (yellow, citrate; red, PEG; blue, avidin; green, anti-MPO) (**A**). Scanned images and colorimetric signal S obtained from reservoirs (top, blue) and polypropylene layer of facemask (bottom, red) before assay (**B**), after nanoparticle transfer (**C**), and after washing away excess reagents (**D**). Facemasks were sprayed with 30 µg·mL^−1^ MPO. Control experiments were performed with reservoirs filled with BSA-coated nanoparticles. Error bars are standard deviation of three independent experiments. A: absorbance; λ (nm): wavelength; S refers to colorimetric signal of colored dots quantified using ImageJ.

Figure 2 shows experiments aimed at characterizing the proposed detection system. The colorimetric detection mechanism relies on specific biorecognition mediated by nanoparticle-labeled antibodies. The specific interaction between antigens and antibodies enables the targeted identification of the analyte, in this case, MPO. Furthermore, the incorporation of gold nanoprobes as labeling agents significantly enhances the sensitivity of the immunoassay, owing to the unique optical properties derived from the localized surface plasmon resonance (LSPR) exhibited by noble metal nanoparticles

Nanoparticles were modified with PEG by means of strong thiol–gold interactions. The resulting PEGylated nanoparticles have demonstrated long-term stability under a range of environmental conditions [26,27,28]. Avidin was attached to the nanoparticles by means of irreversible amide bonds between carboxylate groups on the nanoprobes and amine groups on the protein. Antibodies were then grafted to the nanoparticles by strong biotin–avidin interactions. The optimization of the antibody concentration around the nanoparticles is detailed in Figure S1 in the Supplementary Materials. Of note, extinction spectra in Figure 2 show slight red-shifts and no widening after each modification step, which is in agreement with the spectral properties of non-aggregated nanoparticle dispersion. Furthermore, images in Figure 2B–D show red-colored spots, which suggests that the nanoprobes do not aggregate even after drying them in the polyelectrolyte-filled paper reservoir.

In Figure 2A, the LSPR of citrate-capped gold nanoparticles (Figure 2A, yellow series) red-shifts upon modification with poly(ethylene glycol) (PEG) molecules ending in thiol and carboxylate groups (Figure 2A, red series), indicating successful coating of the colloids with the polymer. The LSPR shifts again after binding avidin to the carboxylate group through peptide bond formation via 1-Ethyl-3-(3-dimethylaminopropyl)carbodiimide hydrochloride (EDC) and N-Hydroxisulfosuccinimide (sulfo-NHS) coupling (Figure 2A, green series). However, no further shift is observed following the addition of biotinylated anti-MPO (Figure 2A, blue series), likely due to the increased distance between the antibodies and the surface of the nanoparticles after modification with PEG and avidin. To verify the presence of antibodies on the nanoprobes, a functional assay was conducted to evaluate the specific recognition of MPO. Nanoparticles functionalized with either anti-MPO or biotinylated BSA (as control) were tested on facemask samples sprayed with 30 µg·mL^−1^. Figure 2B shows images of the nanoparticle reservoirs and facemasks prior to transfer. The reservoirs, which contain concentrated gold nanoparticles, display intense coloration and thus produce strong colorimetric signals.

In Figure 2C, colored dots become visible on the facemasks after contact-pressing the paper decals for 5 min. A 46.2 ± 1.7 reduction in colorimetric signal was observed in the reservoirs (blue series), confirming that the nanoparticles were successfully transferred. Importantly, the transferred signal was comparable between anti-MPO and BSA-coated nanoparticles (red series), demonstrating that the transfer efficiency was not influenced by the surface coating. However, as shown in Figure 2D, most of the BSA-coated (control) nanoparticles were removed during the washing step, whereas anti-MPO nanoprobes remained bound to the facemask surface, generating higher colorimetric signals. These results confirm the specific recognition of MPO by the antibody-functionalized nanoparticles and thereby validate the successful antibody conjugation to the nanoparticle surface. They also confirm that antibodies do not detach completely after storing antibody-decorated nanoparticles in the paper reservoirs, since the nanoprobes yield higher signals than the control nanoparticles for detecting MPO.

After confirming the specific recognition of MPO by the nanoprobes, we evaluated whether they could yield dose-dependent signals. To this end, MPO solutions at different concentrations were prepared in PBS and sprayed onto a piece of facemask using a spray bottle, which emulates aerosol trapping by the facemask [16]. MPO concentrations ranged from 0 to 3 · 10^1^ µg·mL^−1^, covering the typical concentration range reported in other respiratory samples such as sputum [29,30]. Anti-MPO nanoparticles were then contact-transferred from a paper decal, as described in Figure 2.

**Figure 3:**
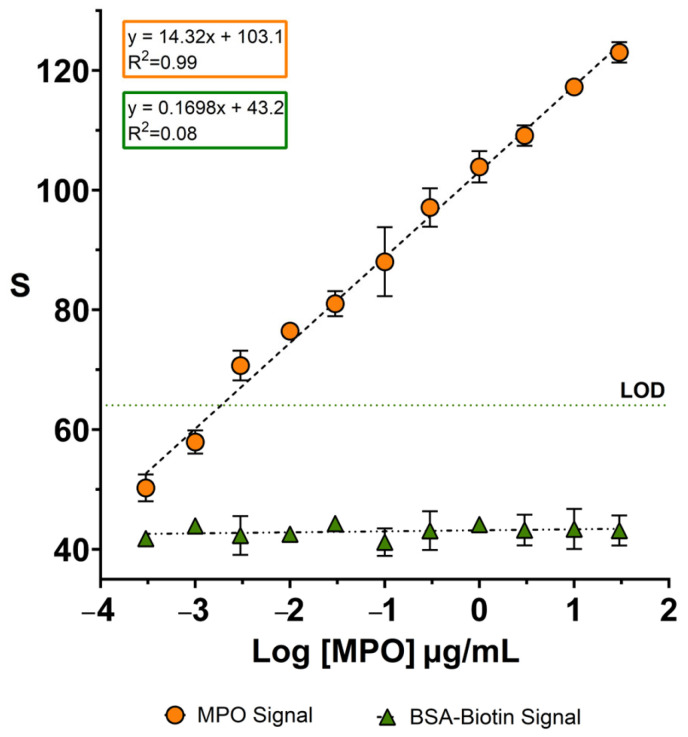
A calibration plot for detecting MPO sprayed on facemasks using nanoparticles coated with anti-MPO (orange dots) or BSA (green triangles). The dotted line indicates the limit of detection (3σ criterion). Error bars are the standard deviation from measuring three independent samples. The symbols without visible error bars indicate that the standard deviation is smaller than the symbol size. LOD: limit of detection. S refers to the colorimetric signal of the colored dots quantified using ImageJ.

As shown in Figure 3, anti-MPO nanoparticles produce a clear dose-dependent signal between 3 · 10^−4^ and 3 · 10^1^ µg·mL^−1^. The calibration curve followed the linear regression equation y = 14.32x + 103.1, with a coefficient of determination r^2^ = 0.9940 and a slope *p*-value of *p* < 0.0001, indicating excellent linearity (raw data shown in Table S1). In contrast, BSA-coated nanoparticles yield a very low and concentration-independent signal, described by the calibration equation y = 0.1698x + 43.19, with an r^2^ of 0.08, and a slope *p*-value of *p* = 0.3904, indicating no significant dose–response relationship. These results confirm the high specificity of the anti-MPO nanoprobes for their target. The dotted line in Figure 3 marks the limit of detection (LOD), defined as the signal of the blank plus three times the standard deviation (3σ criterion, 99% confidence). The first point in the calibration plot above this signal is 3 · 10⁻^3^ µg·mL^−1^.

Our detection range spans from 3 · 10^−4^ to 3 · 10^1^ µg·mL⁻^1^, covering a broad spectrum of MPO values described in COPD patients. For instance, studies using sputum samples from stable COPD patients reported MPO levels between 0 and 1.6 · 10^4^ pg/mL [31], 2.4 and 6.1 ng/mg [32], and 9 · 10^6^ and 2.3 · 10^7^ pg/mL [33]. In exacerbated patients, values ranges from 2.5 · 10^3^–1.6 · 10^4^ pg/mL have been reported [31]. Even in healthy individuals, sputum MPO concentrations between 1 · 10^6^ and 1.6 · 10^7^ pg/mL have been reported [33]. Other samples such as PEx [5] or EBC [10] report much lower values, often in the pg/mL or mU/mL range; however, these values are highly influenced by the sampling technique, dilution, and analytical method applied.

It is important to note that our assay was optimized to detect MPO directly from exhaled air collected using surgical masks—a novel and non-invasive matrix. Nevertheless, our lower detection limit (3 · 10⁻^3^ µg/mL) was comparable to values reported for sputum analysis (Table S2), and the assay’s sensitivity enables the detection of concentration increases consistent with those reported in exacerbated patients [31], supporting its potential use in identifying COPD exacerbations.

It should be noted that the LOD shown in Figure 3 may be influenced by experimental factors such as the distance between the spray bottle and the facemask, the volume dispensed per spray, and the uniformity of aerosol deposition. While the use of a spray bottle provides a practical and reproducible method to emulate aerosol trapping under controlled laboratory conditions, it does not fully replicate the complexity of real respiratory aerosol generation. Parameters such as particle size distribution, humidity, and airflow dynamics during breathing differ substantially from those produced by manual spraying. Therefore, while this in vitro approach provides a reliable platform for evaluating sensor performance and establishing detection thresholds, future studies using clinical breath samples will be necessary to validate the system under physiological conditions.

After demonstrating that the assay can specifically detect the target biomarker, we next assessed its ability to discriminate different levels of airway inflammation in patients with COPD. A total of 22 patients diagnosed with COPD were enrolled, and 36.3% (*n* = 8) of them presented with exacerbations at the time of sampling. Spontaneously expectorated sputum samples were obtained from these eight exacerbated patients and processed for culture after confirming that the samples were representative of the distal airways according to standard criteria [23]. Among the eight sputum samples, three pathogens were identified: *Stenotrophomonas melophilia*, *Proteus mirabilis*, and *Moraxella catarrhalis*. The remaining five sputum samples were culture-negative, suggesting that the exacerbations were likely caused by viral infections or non-infectious triggers. Additionally, 21 healthy subjects without COPD were included as controls.

Table 1 presents the baseline and clinical characteristics of the study population, grouped according to the presence of exacerbation (COPD-E), absence of exacerbation (COPD-NE), and patients without COPD (control). COPD patients were significantly older than the control group (72.7 [COPD-E] vs. 69.2 [COPD-NE] vs. 41.4 [control] years old, *p* < 0.001). No statistically significant differences were found among the groups regarding sex, body mass index (BMI), mMRC dyspnea scale, Global Initiative for Chronic Obstructive Lung Disease (GOLD) scale, allergies, bronchiectasis, and colonization by *P. aeruginosa* or other pathogens. These similarities suggest that COPD-NE and COPD-E groups were comparable in terms of disease severity and underlying baseline inflammation. The mean white blood cell (WBC) count was elevated in both COPD groups compared to the control group (10 [COPD-E] vs. 9.7 [COPD-NE] vs. 5.9 [control], *p* < 0.001), as well as the neutrophil count (7.2 [COPD-E] vs. 6.6 [COPD-NE] vs. 3.2 [control], *p* < 0.001), which is consistent with higher levels of systemic inflammation in COPD. Lung function parameters were significantly reduced in both COPD groups compared to the controls (*p* < 0.001), particularly in COPD-E. It is plausible that patients with exacerbations may generate fewer aerosols during breathing, which could reduce the amount of MPO retained on the facemask and potentially lead to false negatives. Further details are provided in Table 1.

**Figure 4:**
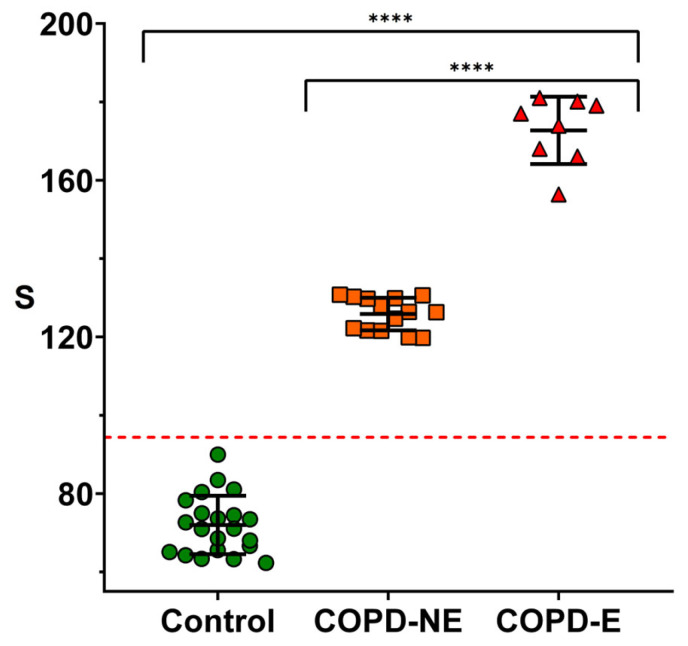
Detection of MPO on masks worn by volunteers using nanoparticles coated with anti-MPO. Healthy individuals (control, green dots), stable COPD patients (COPD-NE, orange squares), and COPD patients during exacerbation (COPD-E, red triangles) are shown. Error bars represent standard deviation for each group. Dotted line indicates limit of detection (3σ criterion). S refers to colorimetric signal of colored dots quantified using ImageJ. Statistical analysis was performed using one-way ANOVA. Significant differences are indicated: “**** *p* < 0.0001”.

Figure 4 shows the results obtained after analyzing the facemasks using the nanoparticle-based immunoassay. All patients experiencing exacerbations (*n* = 8) exhibited significantly higher MPO levels compared to non-exacerbated patients. Importantly, although all exacerbated patients showed elevated MPO levels, only three of them had identifiable pathogens in their sputum culture. This suggests that in the remaining five cases, neutrophilic inflammation may have been triggered by non-bacterial causes, such as viral infections. Notably, facemasks from the COPD-E group showed signals exceeding three times the SD of those from the COPD-NE group, indicating that the proposed method could be used to identify patients undergoing exacerbations. In contrast, MPO immunodetection on facemasks from healthy controls yielded signals that were lower than those observed in all COPD patients. Interestingly, stable COPD patients produced signals that were still significantly higher than the baseline levels in healthy individuals, suggesting that this facemask-based assay could also be useful for characterizing baseline neutrophilic inflammation in the airways of COPD patients.

Further validation studies are needed to explore this possibility, ideally incorporating a broader range of disease severities and age-matched cohorts, as inflammatory status may be influenced by age. Nevertheless, the experiments in Figure 4 demonstrate that MPO detection on facemasks worn by COPD patients is a suitable and non-invasive strategy to assess airway neutrophilic inflammation. These findings lay the groundwork for implementing this approach as a potential tool for the early identification of COPD exacerbations.

## 4. Limitations

This study has some limitations. First, the small sample size, particularly in the COPD-E group, limits the generalizability of the results. Nonetheless, the immunoassay yielded statistically significant differences between clinical groups, supporting its preliminary validity. It is important to emphasize that the primary objective of this pilot study was to evaluate the feasibility of a proof-of-concept test, rather than to conduct large-scale validation. Moreover, the protocol approved by the local ethics committee authorized only the detection of MPO in surgical facemasks, without allowing for gathering additional samples or patient follow-up. Future studies involving larger, multicentric cohorts, repeated measurements, and independent validation groups (along with longitudinal monitoring of MPO levels and clinical outcomes) will be essential to confirm diagnostic performance, ensure reproducibility, and assess the potential utility of the assay for disease monitoring and progression. Second, although the use of a spray bottle for MPO deposition during calibration ensures experimental reproducibility, it does not fully replicate the complex aerosolization patterns associated with natural respiration. As such, the in vitro detection limits reported here should be interpreted with caution and validated in future studies involving exhaled breath samples. Lastly, the sensitivity of the immunoassay is limited, which may affect its ability to distinguish between mild inflammatory states and healthy subjects [10,33,34,35,36], although our sensor can detect at least a threefold increase in MPO concentration. This study did not aim to evaluate the assay’s performance in detecting mild inflammation. Rather, the sensor demonstrated the ability to distinguish between the baseline condition of a healthy subject, that of a patient with stable COPD, and that of a patient undergoing a COPD exacerbation. In future studies, further improvements to the sensor could enable the detection of patients with low-grade inflammation.

Despite these limitations, this work presents notable strengths in the context of gold nanoparticle-based immunoassays. In particular, we introduce a novel implementation of this technology through nanoparticle reservoirs embedded in paper decals, allowing direct and uniform transfer of highly concentrated nanoprobes onto unmodified surgical facemasks. Unlike other systems that require embedded sensors [17,18,19] or electrochemical platforms involving complex instrumentation [37], our decal-based method enables a simple and rapid immunoassay that produces visible results in less than 10 min. Importantly, the assay relies on standard surgical facemasks worn by patients as passive samplers for airway material in a non-invasive manner, particularly relevant for COPD patients who often cannot expectorate [38]. Signal quantification can be performed using widely accessible tools such as desktop scanners or smartphones [21], facilitating the use of this method in primary care settings [9,10,11,12,13]. This combination of simplicity, speed, and non-invasiveness makes our approach a practical and scalable alternative to conventional methods, with strong potential for application in routine clinical monitoring and primary care environments.

## 5. Conclusions

We have introduced a new technology for detecting MPO adhered to surgical facemasks using a nanoparticle-based immunoassay. When applied to facemasks worn by COPD patients, this method identified significantly elevated MPO signals in those experiencing clinically diagnosed exacerbations compared to stable patients. These findings suggest that the proposed method could be used to diagnose COPD exacerbations triggered by neutrophilic inflammation. Furthermore, facemasks worn by healthy subjects yielded MPO signals significantly lower than those from both stable and exacerbated COPD patients. This suggests that the method could be implemented for characterizing basal levels of airway inflammation as well. The analytical procedure involves pressing a paper decal containing the nanoparticle reservoir onto the facemask, applying a washing solution, and analyzing a photograph, steps that could be easily performed in a basic laboratory or automated with a small instrument. This simplified procedure, combined with the simple sampling requirement of wearing a surgical facemask for 30 min, highlights the potential of this approach as a diagnostic tool for airway diseases across different levels of healthcare, from specialized clinics to point of healthcare.

## Figures and Tables

**Table 1:** The baseline characteristics of the studied population.

	COPD-E	COPD-NE	Control	*p*-Value
**Total patients**	8	14	21	
**Age, mean ± SD**	72.7 ± 7.3	69.2 ± 4.7	41.4 ± 5.5	<0.0001 *
**Sex, *n* (%)**				0.15
**Male**	7 (87.5)	8 (57.1)	10 (47.6)	
**Female**	1 (12.5)	6 (42.8)	11 (52.4)	
**Active smoking, n (%)**				0.001 *
**Yes**	1 (12.5)	5 (35.7)	5 (23.8)	
**No ^#^**	7 (87.5)	9 (64.3)	5 (23.8)	
**Never smoker**	0	0	11 (52.4)	
**Smoking index (pack–years), median (IQR)**	50 (50–80)	47.5 (40–71.2)	1.4 (0.7–4)	<0.0001 *
**BMI (kg/m^2^), mean** ** ± ** **SD**	24.5 ± 5	27.3 ± 4.7	24.7 ± 4.5	0.3775
**Dyspnea level (mMRC scale), median (IQR)**	3 (2.2–3)	2 (2–3)	NA	0.3528
**Airflow limitation severity (GOLD), n (%)**				0.6613
**GOLD 2**	1 (12.5)	4 (28.5)	NA	
**GOLD 3**	3 (37.5)	4 (28.5)	NA	
**GOLD 4**	3 (37.5)	4 (28.5)	NA	
**Lung function, mean ± SD**				
**FVC_post_ (% predicted)**	78.8 ± 20.6	67.7 ± 26.8	100.4 ± 15.1	0.0003 *
**FEV1_post_ (% predicted)**	34.8 ± 15.3	41 ± 17.9	104.8 ± 13.7	<0.0001 *
**FEV1/FVC_post_**	32.7 ± 9.4	40.1 ± 9.2	83.7 ± 4.6	<0.0001 *
**DLCO (% predicted)**	46.8 ± 32.3	36.7 ± 10.7	92.2 ± 12.6	<0.0001 *
**PaO_2_ (mmHg)**	66 (66–66)	66.5 (45–79)	NA	0.8473
**PaCO_2_ (mmHg)**	51 (43–59)	45 (44–50)	NA	0.6415
**Laboratory values, mean ± SD**				
**WBC (·10^9^/L)**	10 ± 4.5	9.7 ± 2.6	5.9 ± 1.1	<0.0001 *
**Neutrophils (·10^9^/L)**	7.2 ± 4.9	6.6 ± 2.3	3.2 ± 0.6	<0.0001 *
**Neutrophils (%)**	68.6 ± 15.6	68 ± 9.4	53.9 ± 9.2	0.0004 *
**Eosinophils (·10^9^/L)**	0.1 ± 0.2	0.2 ± 0.1	0.1 ± 0.07	0.4870
**Eosinophils (%)**	2.2 ± 2.5	2.2 ± 1.8	2.6 ± 1.5	0.8045
**C-reactive protein (mg/dL)**	0.09 ± 0.07	1.4 ± 2.1	0.1 ± 0.1	0.1492
**Allergies, n (%)**	0	3 (21.4)	7 (33.3)	0.7041
**Bronchiectasis, n (%)**	1 (12.5)	5 (35.7)	0	0.3512
**PA colonization, n (%)**	1 (12.5)	4 (28.5)	NA	0.6130
**Other pathogen colonization, n (%)**	2 (25)	3 (21.4)	NA	>0.9999
**MPO levels, mean ± SD**	172.7 ± 8.6	125.8 ± 4.2	72 ± 7.5	<0.0001 *

(^#^) Ex-smokers: abstinence for at least last twelve months; (*) significant *p* ≤ 0.01 refers to comparison among three groups. BMI: body mass index; COPD-E: chronic obstructive pulmonary disease patients with exacerbations; COPD-NE: chronic obstructive pulmonary disease patients without exacerbations; DLCO: diffusion capacity for carbon monoxide; FEV1: forced expiratory volume in 1 s; FVC: forced vital capacity; GOLD: Global Initiative for Chronic Obstructive Lung Disease; IQR: interquartile range; mMRC: modified medical research council; NA: not assessed; PA: *Pseudomonas aeruginosa*; SD: standard deviation; WBC: white blood cell.

## Data Availability

Data supporting the findings of this study are not openly available due to reasons of sensitivity and are available from the corresponding author (S.T.) upon reasonable request. Data are located in controlled access data storage at the Pneumology Department of the Hospital Universitario Son Espases, Balearic Islands, Spain.

## Supplementary Materials

The following supporting information can be downloaded at: https://www.mdpi.com/article/10.3390/bios15050323/s1, Figure S1: Testing antibody concentrations for the detection of Myeloperoxidase (MPO). Antibody performance was assessed by comparing signal intensities between negative controls (0 µg/mL MPO, displayed in gray) and samples with 3 µg/mL MPO (displayed in red). Error bars indicate the standard deviation based on three independent experiments; Table S1: Raw data of pixel intensity (PI) and colorimetric signal (S) for MPO detected on facemasks using nanoparticles coated with anti-MPO antibodies. S = integral value of (255 - PI)” and PI was calculated in this way for each spot in the triplicates, and the same procedure was applied to the entire dataset; Table S2: Summary of MPO levels studies in human respiratory samples across different COPD phases. The table includes sample type, disease condition, MPO values (mean or median) and ranges, detection methods used, and references. MPO values are reported as found in the literature. LPS: Lipopolysaccharide endotoxin challenge model; PEx: Exhaled breath particles; EBC: Exhaled breath condensate. References [10,31,32,33,35,36] are cited in the Supplementary Materials.

## Abbreviations

The following abbreviations are used in this manuscript:

COPD	Chronic obstructive pulmonary disease
MPO	Myeloperoxidase
PEG	Poly(ethyleneglycol)
LOD	Limit of detection
Sulfo-NHS	N-Hydroxysulfosuccinimide sodium salt
S	Colorimetric signal
Abs	Absorbance
BSA	Bovine serum albumin
PBS	Phosphate-buffered saline
SH-PEG-COOH	Poly(ethylene glycol) 2-mercaptoethyl ether acetic acid
EDC	1-etil-3-(3-dimetilaminopropil)carbodiimida
PSS	Poly(4-styrenesulfonate) sodium salt
SD	Standard deviation
IQR	Interquartile ranges
LSPR	Localized surface plasmon resonance
NIR	Near-infrared
GOLD	Global Initiative for Chronic Obstructive Lung Disease
COPD-E	Chronic obstructive pulmonary disease patients with exacerbations
COPD-NE	Chronic obstructive pulmonary disease patients without exacerbations
WBC	White blood cell
BMI	Body mass index
DLCO	Diffusion Capacity of Carbon Monoxide
FEV1	Forced expiratory volume in 1 s
FVC	Forced Vital Capacity
mMRC	Modified Medical Research Council
NA	Not assessed
PA	*Pseudomonas aeruginosa*
