# Supplementary material for: A Nanoparticle-Based Immunoassay on Facemasks for Evaluating Neutrophilic Airway Inflammation in COPD Patients
Source: Biosensors (Basel). 2025 May 19;15(5):323. doi: 10.3390/bios15050323 (PMC12110278; doi:10.3390/bios15050323)
Supplement: Supplementary file 1 [file biosensors-15-00323-s001.zip › biosensors-3598102-supplementary.pdf]

Supplementary Materials

A Nanoparticle-Based Immunoassay on Facemasks for Evaluating Neutrophilic Airway Inflammation in COPD Patients

Bartomeu Mestre [1], Nuria Toledo-Pons [2,3], Andreu Vaquer [1,4,*], Sofia Tejada [1,*], Antonio Clemente [5,6],
Amanda Iglesias [2,7], Meritxell López [2,3,7], Ruth Engonga [2,3], Sabina Perelló [2,3], Borja G. Cosío [2,3,7]
and Roberto de la Rica [1,6]

1 Multidisciplinary Sepsis Group, Health Research Institute of the Balearic Islands (IdISBa), 07120 Palma de Mallorca, Spain; bartomeu.mestrec@idisba.es (B.M.); antonio.clemente@idisba.es (A.C.); roberto.delarica@idisba.es (R.R.)
2 Inflamación, Reparación y Cáncer en Enfermedades Respiratorias (I-RESPIRE) Group, Health Research Institute of the Balearic Islands (IdISBa), 07120 Palma de Mallorca, Spain; nuria.toledo@ssib.es (N.T-P.); amanda.iglesias@ssib.es (A.I.); meritxell.lopez@ssib.es (M.L.); ruth.engonga@ssib.es (R.E.); sabina.perello@ssib.es (S.P.); borja.cosio@ssib.es (B.G.C.)
3 Department of Respiratory Medicine, Hospital Universitario Son Espases, 07120 Palma de Mallorca, Spain
4 Department of Chemistry, University of the Balearic Islands, 07122 Palma de Mallorca, Spain
5 Group of Innovation in Immunopathology of Infections (GTERi), Health Research Institute of the Balearic Islands (IdISBa), 07120 Palma de Mallorca, Spain
6 Centro de Investigación Biomédica en Red de Enfermedades Infecciosas, Instituto de Salud Carlos III (CIBERINFEC), 28029 Madrid, Spain
7 Centro de Investigación Biomédica en Red de Enfermedades Respiratorias, Instituto de Salud Carlos III (CIBERES), 28029 Madrid, Spain
* Correspondence: andreu.vaquer@idisba.es (A.V.); sofia.tejada@idisba.es (S.T.)

SECTION 1. Raw Data of Colorimetric Detection of MPO on Facemasks.

Table S1. Raw data of pixel intensity (PI) and colorimetric signal (S) for MPO detected on facemasks using nanoparticles coated with anti-MPO antibodies. S = integral value of (255 - PI)" and PI was calculated in this way for each spot in the triplicates, and the same procedure was applied to the entire dataset

Log [MPO] µg/mL	MPO µg/mL	Pixel Intensity (PI)	Mean	SD	Colorimetric Signal (S)	Mean
**-3.52**	**0.0003**	205.13	204.75	2.24	49.87	50.25
		206.77			48.23	
		202.34			52.66	
**-3.00**	**0.001**	195.57	197.07	1.93	59.43	57.93
		196.40			58.60	
		199.25			55.75	
**-2.52**	**0.003**	186.89	184.32	2.47	68.11	70.68
		184.11			70.89	
		181.95			73.05	
**-2.00**	**0.01**	177.72	178.57	1.04	77.28	76.43
		179.73			75.27	
		178.28			76.72	
**-1.52**	**0.03**	173.79	173.99	2.11	81.21	81.02
		171.98			83.02	
		176.19			78.81	
**-1.00**	**0.1**	168.77	166.97	5.77	86.23	88.03
		160.51			94.49	
		171.62			83.38	
**-0.52**	**0.3**	161.29	157.90	3.21	93.71	97.10
		157.51			97.49	
		154.91			100.09	
**0.00**	**1**	153.65	151.10	2.62	101.35	103.90
		151.23			103.77	
		148.42			106.58	
**0.48**	**3**	147.83	145.88	1.71	107.17	109.12
		145.14			109.87	
		144.67			110.33	
**1.00**	**10**	137.64	137.73	1.37	117.36	117.27
		136.42			118.59	
		139.15			115.86	
**1.48**	**30**	132.46	131.98	1.70	122.54	123.02
		130.09			124.91	
		133.39			121.61	

SECTION 2. Example of MPO Levels in Respiratory Samples from COPD Studies.

Table S2. Summary of MPO levels studies in human respiratory samples across different COPD phases. The table includes sample type, disease condition, MPO values (mean or median) and ranges, detection methods used, and references. MPO values are reported as found in the literature. LPS: Lipopolysaccharide endotoxin challenge model; PEx: Exhaled breath particles; EBC: Exhaled breath condensate.

Respiratory Sample	COPD Phase	MPO Value	MPO Range	Detection Method	Reference
Sputum	Healthy Volunteers (non-smokers)	$3.0 \cdot 10^6$ pg·mL^{-1}	-	ELISA	[1]
Sputum	Healthy Volunteers (smokers)	$2.5 \cdot 10^6$ pg·mL^{-1}	-	ELISA	[1]
Sputum	Stable	$5.2 \cdot 10^6$ pg·mL^{-1}	0 - $1.6 \cdot 10^4$ pg·mL^{-1}	ELISA	[1]
Sputum	Exacerbated	-	$2.5 \cdot 10^3$ - $1.6 \cdot 10^4$ pg·mL^{-1}	ELISA	[1]
Sputum	Healthy Volunteers	$(6.58 \pm 1.87) \cdot 10^4$ pg·mL^{-1}	-	ELISA	[2]
Sputum	Stable	$(5.74 \pm 1.12) \cdot 10^5$ pg·mL^{-1}	-	ELISA	[2]
Sputum	Healthy Volunteers	$2.0 \cdot 10^5$ pg·mL^{-1}	-	Electrochemical immunosensor	[3]
Sputum	Stable	$9.1 \cdot 10^6$ pg·mL^{-1}	2.4-6.1 ng/mg	Electrochemical immunosensor	[3]
Sputum	Healthy Volunteers (non-smokers)	$3.0 \cdot 10^6$ pg·mL^{-1}	$2.0 \cdot 10^6$ - $1.6 \cdot 10^7$ pg·mL^{-1}	ELISA	[4]
Sputum	Healthy Volunteers (smokers)	$4.0 \cdot 10^6$ pg·mL^{-1}	$1.0 \cdot 10^6$ - $1.5 \cdot 10^7$ pg·mL^{-1}	ELISA	[4]
Sputum	Stable	$1.2 \cdot 10^7$ pg·mL^{-1}	$9.0 \cdot 10^6$ - $2.3 \cdot 10^7$ pg·mL^{-1}	ELISA	[4]
Pex	Healthy Volunteers	$1.0 \cdot 10^{-1}$ pg·mL^{-1}	$1.0 \cdot 10^{-1}$ - $8.0 \cdot 10^{-1}$ pg·mL^{-1}	LPS	[5]
EBC	Healthy Volunteers	3 mU/mL	0-9 mU/mL	Immunoassay	[6]
EBC	Exacerbated	20 mU/mL	0-70 mU/mL	Immunoassay	[6]

SECTION S3. Optimization of anti-MPO around the nanoparticles.

The ideal antibody concentration for coating nanoparticles was determined by analyzing signal responses across a range of antibody concentrations: 12.5, 25, 50, 100, and 200 μg·mL^{-1}. For each concentration, signal intensity was compared between a negative control (0 μg·mL^{-1} of MPO) and a positive control (3 μg·mL^{-1} of MPO). The optimal concentration was defined as the one that produced the greatest difference in signal between these two conditions. In the case of Myeloperoxidase (MPO) detection, 25 μg·mL^{-1} yielded the highest signal distinction and was selected as the optimal antibody concentration.

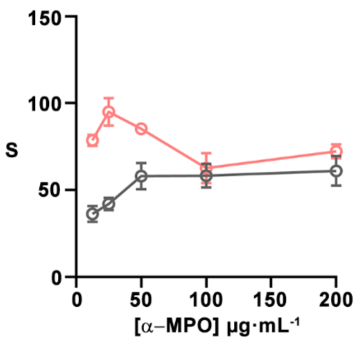

Figure S1. Testing antibody concentrations for the detection of Myeloperoxidase (MPO). Antibody performance was assessed by comparing signal intensities between negative controls (0 μg/mL MPO, displayed in gray) and samples with 3 μg/mL MPO (displayed in red). Error bars indicate the standard deviation based on three independent experiments.

REFERENCES

1. Mulvanny, A.; Pattwell, C.; Beech, A.; Southworth, T.; Singh, D. Validation of Sputum Biomarker Immunoassays and Cytokine Expression Profiles in COPD. *Biomedicines* **2022**, *10*, 1949, doi:10.3390/biomedicines10081949.
2. Guo, X.; Deng, N.; Chen, Q.; Yu, H.; Ding, X.; Hu, S.; Nie, H. Application of Inflammatory Markers in Induced Sputum in Stable Chronic Obstructive Pulmonary Disease Patients with Positive Bronchodilation Tests. *CURR MED SCI* **2019**, *39*, 560–567, doi:10.1007/s11596-019-2074-7.
3. Gutiérrez-Capitán, M.; Sanchís, A.; Carvalho, E.O.; Baldi, A.; Vilaplana, L.; Cardoso, V.F.; Calleja, Á.; Wei, M.; de la Rica, R.; Hoyo, J.; et al. Engineering a Point-of-Care Paper-Microfluidic Electrochemical Device Applied to the Multiplexed Quantitative Detection of Biomarkers in Sputum. *ACS Sens* **2023**, *8*, 3032–3042, doi:10.1021/acssensors.3c00523.
4. Iwamoto, H.; Gao, J.; Koskela, J.; Kinnula, V.; Kobayashi, H.; Laitinen, T.; Mazur, W. Differences in Plasma and Sputum Biomarkers between COPD and COPD-Asthma Overlap. *Eur Respir J* **2014**, *43*, 421–429, doi:10.1183/09031936.00024313.
5. Holz, O.; Müller, M.; Carstensen, S.; Olin, A.-C.; Hohlfeld, J.M. Inflammatory Cytokines Can Be Monitored in Exhaled Breath Particles Following Segmental and Inhalation Endotoxin Challenge in Healthy Volunteers. *Sci Rep* **2022**, *12*, 5620, doi:10.1038/s41598-022-09399-z.
6. Tateosian, N.L.; Costa, M.J.; Guerrieri, D.; Barro, A.; Mazzei, J.A.; Eduardo Chuluyan, H. Inflammatory Mediators in Exhaled Breath Condensate of Healthy Donors and Exacerbated COPD Patients. *Cytokine* **2012**, *58*, 361–367, doi:10.1016/j.cyto.2012.03.006.
